# Bioclimatic modeling in the Last Glacial Maximum, Mid-Holocene and facing future climatic changes in the strawberry tree (*Arbutus unedo* L.)

**DOI:** 10.1371/journal.pone.0210062

**Published:** 2019-01-09

**Authors:** Maria Margarida Ribeiro, Natália Roque, Sílvia Ribeiro, Catarina Gavinhos, Isabel Castanheira, Luís Quinta-Nova, Teresa Albuquerque, Saki Gerassis

**Affiliations:** 1 Departamento de Recursos Naturais e Desenvolvimento Sustentável, Instituto Politécnico de Castelo Branco, Escola Superior Agrária, Castelo Branco, Portugal; 2 Forest Research Centre, School of Agriculture, University of Lisbon, Tapada da Ajuda, Lisbon, Portugal; 3 Centro de Biotecnologia de Plantas da Beira Interior, Quinta da Senhora de Mércules, Castelo Branco, Portugal; 4 Centro de Recursos Naturais, Ambiente e Sociedade (CERNAS) - Instituto Politécnico de Castelo Branco, Castelo Branco, Portugal; 5 Centro de Investigação em Agronomia, Alimentos, Ambiente e Paisagem (Linking Landscape, Environment, Agriculture and Food), Instituto Superior de Agronomia, Tapada da Ajuda, University of Lisbon, Lisbon, Portugal; 6 Instituto Politécnico de Castelo Branco, Escola Superior de Tecnologia, Castelo Branco, Portugal; 7 Department of Natural Resources and Environmental Engineering, Vigo University, Lagoas, Marcosende, Vigo, Spain; Pacific Northwest National Laboratory, UNITED STATES

## Abstract

Increasing forest wildfires in Portugal remain a growing concern since forests in the Mediterranean region are vulnerable to recent global warming and reduction of precipitation. Therefore, a long-term negative effect is expected on the vegetation, with increasing drought and areas burnt by fires. The strawberry tree (*Arbutus unedo* L.) is particularly used in Portugal to produce a spirit by processing its fruits and is the main income for forestry owners. Other applications are possible due to the fruit and leaves’ anti-oxidant properties and bioactive compounds production, with a potential for clinical and food uses. It is a sclerophyllous plant, dry-adapted and fire resistant, enduring the Mediterranean climate, and recently considered as a possibility for afforestation, to intensify forest discontinuity where pines and eucalypts monoculture dominate the region. To improve our knowledge about the species’ spatial distribution we used 318 plots (the centroid of a 1 km^2^ square grid) measuring the species presence and nine environmental attributes. The seven bioclimatic variables most impacting on the species distribution and two topographic features, slope and altitude, were used. The past, current and future climate data were obtained through WorldClim. Finally, the vulnerability of the strawberry tree to the effects of global climate change was examined in the face of two emission scenarios (RCP 4.5 and 8.5), to predict distribution changes in the years 2050 and 2070, using a species distribution models (MaxEnt). The reduction of suitable habitat for this species is significant in the southern regions, considering the future scenarios of global warming. Central and northern mountainous regions are putative predicted refuges for this species. Forest policy and management should reflect the impact of climate change on the usable areas for forestry, particularly considering species adapted to the Mediterranean regions and wildfires, such as the strawberry tree. The distribution of the species in the Last Glacial Maximum (LGM) and Mid-Holocene (MH) agrees with previous genetic and paleontological studies in the region, which support putative refuges for the species. Two in the southern and coastal-central regions, since the LGM, and one in the east-central mountainous region, considered as cryptic refugia.

## Introduction

The Mediterranean region suffers from a high increase in temperature are now ca. 1.5-fold than during 1880–1920, compared to the other regions in the world. The impact of climate change in this region should be taken into consideration the design of land-use policies and, the conservation of genetic resources (>2°C above preindustrial levels) [1].

The prediction of the main impacts on the forest in Portugal, due to climate change, suggest a trend on species migration, from south to north and from the inner lands to the coastal areas. Moreover, under this scenario, forests may even disappear from what are now the drier areas (e.g. southern interior region). The risk of wildfires will increase in a hotter and drier climate and may further be increased by the higher accumulation of high flammability biomass during the fire season. The impact on forest economics can be extremely severe: decreased productivity, increased fire risk, pests and diseases’ risk can turn forest investment unattractive, resulting in increasing forest abandonment [2, 3].

Regarding adaptation measures, the need to establish adaptive forest management is crucial. Management should incorporate the emerging knowledge on climate change and be considered as a process of continuous learning. Thus, monitoring, and assessment components are fundamental in this context. Simultaneously, silviculture techniques should consider climate change (e.g. [4]). Forest policies and their instruments should be aware of climate change, particularly in which concerns forest fire prevention [2]. Indeed, wildfires’ burned area is predicted to increase from 3 to 5-fold until the end of the century in the Mediterranean regions [5]. The burned area in wildfires is in Portugal higher than in any other southern European country and this phenomenon has a tendency to increase [6, 7]: e.g. in the year of 2017 more than 400 thousand ha were burned. Additionally, Portuguese forests have been exposed to extensive human impact, which caused the replacement of oak-climax forests by shrubland and agriculture [8]. Habitat loss and agriculture intensification have fragmented and degraded natural areas, which can be further intensified under climatic and anthropogenic changes.

Predicting and understanding how environmental changes impacted and will impact, the species’ composition and distribution, is a critical issue for biogeography and biological conservation. Ecological Niche Models are widely used to generate information about abiotic preferences and tolerances of species (i.e., the existing fundamental niche *sensu* Peterson et al. [9]) and, hence, to evaluate potential distributions in different time periods, in past and future potential scenarios, using ecological conditions estimated with present information [10].

In the study, we aimed at studying a typically Mediterranean species widely distributed in the country and in the Mediterranean area, the strawberry tree (*Arbutus unedo* L.). By modeling it is possible to evaluate the ecological past and present species distribution range, to reveal the impact of environmental features on the strawberry tree’s habitat distribution, and to further evaluate the ecological niche’s change using contrasting global warming scenarios.

We also investigated whether the climate, during the Last Glacial Maximum (~ 22 000 BP) and the Mid-Holocene (~ 6 000 BP) supports the strawberry tree refugia, identified by paleontological and phylogeographical data ([6] and references therein). Ecological niche modeling allowed the evaluation of the ecological requirements of the species, based on the knowledge of existences and enabling the prediction of its distribution in projected future and past scenarios. This approach help in identifying important areas for conservation and those potentially endangered in the future, due to the global warming impact.

This species is an evergreen small tree or shrub, native to the Mediterranean region and Western Europe, and belongs to the Ericaceae family and to sclerophyllous and laurel-like flora. The species’ distribution is mainly throughout areas where frost and summer dryness are milder, especially in coastal and inland regions. Moreover, the strawberry tree is ecologically versatile, growing on a wide variety of soil conditions, from siliceous to calcareous (except waterlogged soils) [11, 12]. Additionally, the *A*. *unedo* plants are fire or graze resistant, since they can easily resprout [13]. However, they are not able to successfully reproduce and compete when shaded [11]. The strawberry tree develops from sea level up to 1 200 m, in conditions that are edaphically unsuitable for woodland and where the vegetation is restricted to shrub communities. Under these conditions *A*. *unedo* can compete, successfully, with other plants [11, 14]. Moreover, the strawberry tree is unfavoured in areas with lower dry ombrotype, as reported by Monteiro-Henriques et al. [15]. Finally, is important to stress the *A*. *unedo* presence in central Portugal during the LGM period [16] and in the Mid-Holocene [17], due to fossil charcoal evidences. The habitat suitability for the strawberry tree, was defined using a niche model software, Maxent [18–20], through analysis of the nowadays environmental envelope existing in the region. Afterward, the distribution of the species using contrasting global warming scenarios for the XXI century, the Last Glacial Maximum and Mid-Holocene was undertaken, aiming to explore the effects of climate changes on the species distribution and the predicted relative abundance, for future and past scenarios.

Our research’s main objectives were: i) to understand the environmental covariates for species occurrences, and ii) to evaluate the coupled temporal dynamics, for climate and vegetation in contrasting scenarios to predict potential distributions’ changes, in Last Glacial Maximum and Mid-Holocene and in four future global warming scenarios.

## Material and methods

### Species data acquisition

To investigate the interactions between the species’ distribution and the related climate factors, a 90 425 plots dataset (centroids of a 1-Km^2^ grid), including 318 plots where the species is present, was used (S1 Table). We used three data sources for the species distribution (presence): 1) the 2006 Portuguese National Forest Inventory (http://www.icnf.pt/portal/florestas/ifn), 85 plots; 2) information retrieved from the previous study on genetic structure [6], 54 plots; and 3) the remaining plots from the SIVIM (Sistema de Información de la Vegetación Iberica y Macaronésica) database (http://www.sivim.info/sivi/).

### Environmental data

The altitude was derived from the country Digital Elevation Model (DEM) provided by the Global Multi-resolution Terrain Elevation Data 2010 [21], with a 30 seconds resolution (1 km^2^). The slope was computed through Spatial Analysis extension and Surface Analysis tool in the ArcGIS Desktop 10.3 software [22], using the slope algorithm, in degrees, as described by Burrough and McDonell [23].

Climatic conditions exert a strong control over the species’ geographic distribution [24], therefore, 21 current conditions climate variables (t_max_, t_min_, BIO1 to BIO19, S2 Table) were downloaded from the WorldClim database version 1.4 [25], corresponding to the highest resolution (30 seconds resolution, ~ 1 x 1 km), except for the LGM (2.5 minute resolution, ~ 5 x 5 km). For the LGM a resample was made to a 1 km^2^ resolution, but not interpolated, meaning that the resolution was 5 x 5 km. For the current conditions, interpolations of observed data, representative of 1960–1990, were used. The Extract by Mask tool within ArcGI Desktop 10.3 software was used to retrieve the layers of Portugal.

The obtained matrix was used as input to the construction of a Bayesian Network (BayesiaLab v7.0), aiming for the detection of the keenest indicators for the strawberry tree presence. The definition of the associated climatic variables, was performed using as decision threshold a *p-value* inferior of 1% and the higher scores obtained in the Mutual Information analysis (MI), a metric widely used in information theory [26], with relative significance (RS) above 20% (S3 Table, our unpublished data) [27]. We retained seven bioclimate attributes: the t_max_ (monthly average maximum temperature), the t_min_ (monthly average minimum temperature), the BIO1 (annual mean temperature), the BIO2 (mean diurnal range [mean of monthly (max. temp.-min. temp.)]), the BIO5 (maximum temperature of warmest month), the BIO9 (mean temperature of driest quarter) and the BIO15 (precipitation seasonality—coefficient of variation). This set of climatic attributes was then used as the best predictor variables for the species’ presence in the subsequent modeling. We have included two topographic attributes, altitude and slope, since according to the literature they have an important impact on the strawberry tree’s survival [11, 14]. Indeed, the species depends on the presence of habitats where the vegetation is often dominated by shrub communities, conditions under which it can successfully compete with other plants [11, 14].

Afterwards, the data from the selected climate attributes were downloaded from the WorldClim site, for the future and for the past, 22 000 BP (LGM) and 6 000 BP (MH), (downscaled GCM, built on the Community Climate System Model, United States of America version 4 (CCSM4) General Circulation Model (GCM)) from CMIP5 (Coupled Model Intercomparison Project) [28]. For the future conditions, we have considered two Representative Concentration Pathways (RCPs) scenarios, RCP 4.5 and RCP 8.5, where the numbers refer to the radiative forcing measured in watts per square meter (Wm^-2^) [29], fitted for two future time slices (2050 and 2070). The RCP 4.5 and RCP 8.5 scenarios were selected as they cover a wide range of anthropogenic driving.

### Model generation

The potential strawberry tree habitat was modeled using the software package Maxent version 3.4.1, a maximum entropy niche model software that performs well in presence-only data, for species or communities [19, 30, 31], in comparison with other presence-only models [32, 33].

The model for a specific species is determined from: 1. a set of environmental or climate layers; 2. a set of georeferenced samples where the species presence was observed. The model expresses the species’ suitability, within each grid cell, as a function of the chosen attributes. High computed value indicates that the considered grid cell is predicted to be suitable for that species. The computed model is a probability distribution over all the grid cells. The chosen distribution corresponds to the one showing the maximum entropy and where the accepted likelihood score is equal to the average over samples, for each feature (derived from the environmental layers) [19].

The MaxEnt software allowed the spatial interpolation using the 318 presence experimental points. The model was trained using 75% of species samples, selected randomly, and the model’s performance was evaluated by predicting the remaining 25% species presence. We, thus, created a niche model for the present-day spatial distribution of strawberry tree in the training region (Portugal), with a set of 10 000 background points randomly placed over the region, which was further projected in the past (LGM and MH) and in the future time frames (2050 and 2070). According to existing information we assumed that the ecological requirements of *A*. *unedo* have remained similar over the last climatic cycles [34].

We generated the cloglog (complementary log-log) Maxent output for all models to estimate the probability of presence, as advised by Phillips et al. [31]. We run different regularization values to test the smoothness of the variable curves, which showed no substantial differences and we used the default regularization multiplier equal to 1. Additionally, the following options were selected: 1. initial random seed for each iteration; 2. remotion of duplicate presence records; 3. data plotting; 4. 1 500 maximum iterations. This last setting was conservative enough to allow adequate algorithm convergence and performance [31].

Using the MaxEnt software version 3.4.1, the variables’ importance was fitted with the correspondent contribution for the prediction model, in percentage; higher contribution higher impact in the species predicting the occurrence of the species. The accuracy of the obtained Maxent models were evaluated using the Area Under the Curve (AUC) of the Receiver Operating Characteristic (ROC) plot, which is a widely known measurement of the discriminatory capacity of classification model. A model will be considered more accurate to data discriminating than a random one, if the curve lies above the diagonal of no discrimination, i.e. which corresponds to an AUC higher than 0.5 ([35] and references therein). The AUC values > 0.75 correspond to high discrimination performances [36]. Sensitivity was used to calculating the AUC score (i.e. the proportion of testing presences records predicted present) of a model. To validate and to test models’ performance we run each dataset (for the 6 scenarios described below) using bootstrapping after 100 replications, to generate mean images and spatial uncertainty (standard deviation).

## Results

### Models evaluation and variables contributions

**Fig 1:**
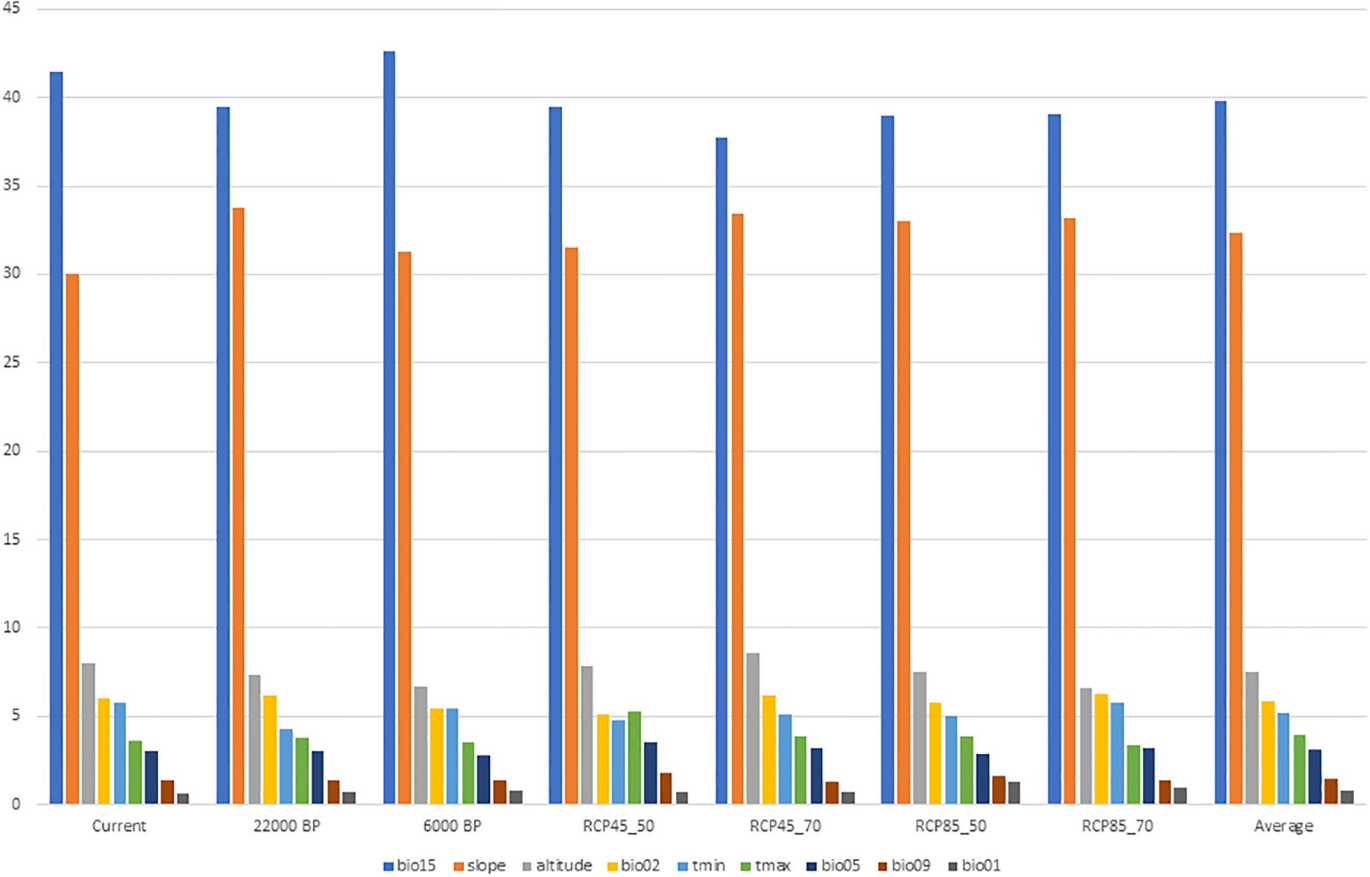
Estimates of the relative contributions, in percentage, of the environmental variables to the Maxent models, fitted to current and projected to past and future climates, including average overall. For each estimate, Maxent keeps track of the environmental variables contributions to the model ([20] for details).

All models were statistically more robust than the random one (AUC = 0.5), with an average AUC of 0.764 ± 0.015. The environmental variables, on average with the highest relative contributions to the Maxent models’ predictions, were the precipitation seasonality (BIO15: 40%), the slope (32%), the altitude (8%), the mean diurnal range (BIO2: 6%), and the monthly average minimum temperature (t_min_: 5%) (Fig 1).

### Habitat niche modeling in present and future conditions

**Fig 2:**
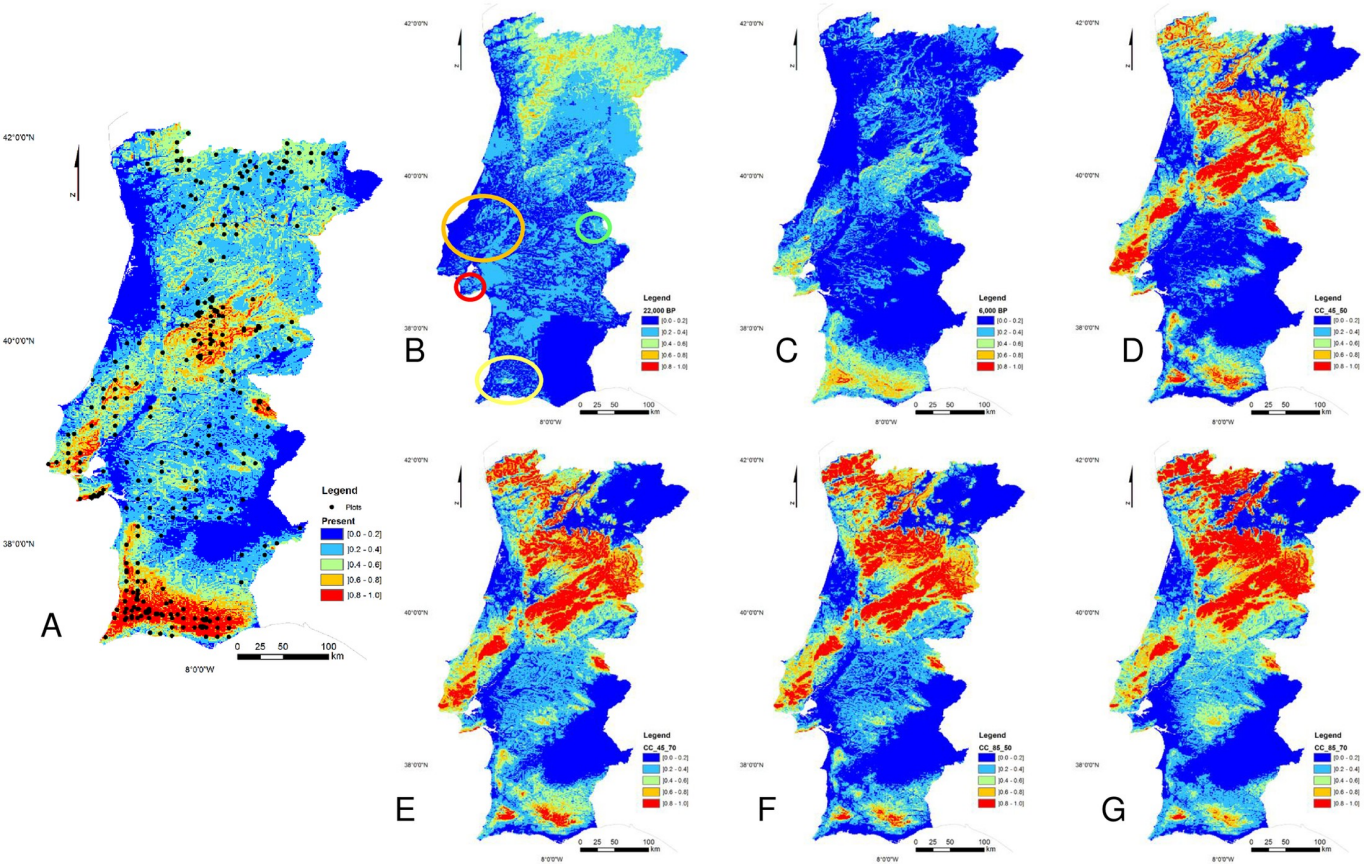
Representation of the Maxent models for the *A*. *unedo* habitat suitability predictions (average over replicated runs). Warmer colors show areas with better-predicted conditions. Black dots show the species presence locations. (A) Present (current climate conditions). (B) Last Glacial Maximum, 20 000 BP. (C) Mid-Holocene, 6 000 BP. (D) Future 2050, RCP 4.5. (E) Future 2070, RCP 4.5. (F) Future 2050, RCP 8.5. (G) Future 2070, RCP 8.5. See text for details.

The differences in habitat suitability, between current and future conditions were compared. The predicted results revealed a reduction of the species’ potential area, in the southern regions and, a clear increase in the northern spots, in the future scenarios (Fig 2A, 2D, 2E, 2F and 2G). Considering a probability of occurrence of the species >20%, until 2050, for the RCP 4.5 and the RCP 8.5 scenarios, the species’ habitat suitability area, had a 20% and 11% reduction, respectively, compared to the nowadays scenario (Fig 2A, 2D and 2F). Until 2070, the habitat suitability area is expected to have a reduction of 15% and 12%, for the same RCP scenarios, respectively (Fig 2A, 2E and 2G).

Considering the impact of the global warming scenarios on the strawberry tree habitat suitability distribution, a trend is observed towards north, where climate is presently cooler and wetter. The regions with the highest probability for the species presence (in red, Fig 2F and 2G), in 2050 and 2070 worst warming scenarios (RCP 8.5), the mountainous regions, become a clear refuge/migration area for the species. Indeed, the northern areas, with the highest species potential presence (>60%), in all future scenarios, have high altitudes (Fig 2D, 2E, 2F and 2G and S2A Fig).

The species decrease in the southern region with the average temperature increase, and the Tagus river becomes a clear border for the species presence in the country. Indeed, the reduction of habitat suitability for this species south of the Tagus river, is very clear (37%) in the best warming scenario (RCP 4.5) for 2050, considering a probability for the species occurrence > 20%, while in the northern region is observed a reduction under the 8%. Mountainous regions are predicted as keen refuges for this species, though also suitable for other forest species and farming, making habitat sharing problematic.

The changes in precipitation seasonality will progressively increase towards 2070 and from southwest to northeast, in both warming scenarios (RCP 4.5 and 8.5), the year of 2070 being the worst for the RCP 8.5 (S1G Fig BIO15). The strawberry tree seems to be better developed in medium-low high (60–67%) precipitation variability (see S1A Fig BIO15 and S3A Fig), but extremely sensitive to very high variation in precipitation seasonality and less advantaged with the future predictions, particularly in the Tagus river’s southern bank.

The slope gains impact in the species presence, avoiding valleys and flat regions (S3B Fig). This species is primarily a plant of shrub communities, commonly developed in steep rocky hillsides or cliffs. For similar reasons, altitude also impacts on the species distribution, increasing sharply to 500 m and smoothly decreasing afterward (S3C Fig). The species seems to be better adapted to mean diurnal range values from 7 to 10°C (S3D Fig), where the values grow from littoral to inland, considering future warming scenarios, and the lower values are found in coastal regions, which might be explained by the preferred species oceanity (S1A, S1D, S1E, S1F and S1G Fig BIO2). When the average minimum temperature increases above 12°C the species habitat suitability decreases sharply (S3E Fig) and, in the future warming scenarios, this threshold is quickly growing northwards (S1D, S1E, S1F and S1G Fig t_min_).

### Contemporary and historical climatic distribution predictions

During the LGM large ice sheets covered high latitude Europe and North America, and the sea levels stood some 120–130 m under the level observed today [37]. Thus, the physiography of Portugal in the LGM had a longer coastline, compared with today’s boundaries, with area now under the sea. Thus, the resulting potential species distribution model in the present was projected onto the climate conditions prevailing during the Last Glacial Maximum, therefore the today’s boundaries were kept.

The past scenario Last Glacial Maximum had much less suitable area for *A*. *unedo* compared to present conditions, 85% of the total area had a probability of presence inferior to 40%, and only 1% with a probability higher than 60%. Nevertheless, a probability between 80 and 100% existed, though extremely low (0.000015%). The area with higher probability of the species presence is found in the northern part of the country (Fig 2B) and, also, with lower probability, in the east-central mountain region (light green). Some light green spots are found in the coastal central area (north from Lisbon, Fig 2B orange circle) and in the Setubal Peninsula (Arrábida region, Fig 2B red circle), an area protected by the dominant winds. Another light green area was found in the Serra de São Mamede (Fig 2B green circle) mountain range, the highest summit south of the Tagus river, and in the southwest in the Serra de Monchique (Fig 2B yellow circle).

The Mid-Holocene output was compared with the probability’s distribution of the present-day environmental conditions and revealed that 63% of the total area has a probability inferior to 20%, for the species presence. Only in a very small area, 8%, is possible to observe a 40–80% probability slice for the species existence. In Fig 2C, we observe a wide median-high probability for the species to occur, in the southern area of the country (orange and light green) and in the coastal central area (Lisbon region). Another location where a noteworthy probability for the species occurrence (20 to 60%) is situated in the east-central mountain region (Serra da Estrela—light blue and green area).

## Discussion

### Habitat change predictions

Based on the results of this study, two environmental variables have a clear influence on the habitat suitability of the strawberry tree, one bioclimatic feature, the precipitation seasonality (BIO15) and the second one, a topographical feature, the slope. The species revealed high vulnerability for low or very extreme precipitation events, as well as, valleys and flat habitats. Indeed, the strawberry tree is primarily a plant adapted to cliffs, broken rocky slopes and rock outcrops, which are edaphically unsuitable for woodland, and where the vegetation is limited to shrub communities. Additionally, *A*. *unedo* is a typical oakwood fringe species, included in the succession steps conducting to climax oakwood, without coping with shading [11]. According to Torres et al. [12], this plant is typical of Mediterranean sclerophyllous and laurel-like vegetation, that appears protected in thermal gorges and in coastal areas with steep topography with a predilection for Mediterranean climate under oceanic influence. In the Iberian Peninsula, it appears in all the biogeographical provinces of the Mediterranean region, although more extensive examples exist in those regions where oceanicity is important. The presence of oligotrophic soils and a high level of rainfall, decreasing in numbers towards inland regions, characterized these continentalized areas. The altitude is also a shaping variable for the species’ distribution but to a lesser extent. The strawberry tree grows from sea level up to 1200 m [11]. Takrouni and colleagues [38] indicate the species’ preference for altitudes ranging from 400 to 1200 m. The results of the current study corroborate the literature information, but altitudes around 500 m are preferred.

The results obtained in the current study for the best scenario, projection RCP 4.5 in 2050, intensified in the RCP 8.5 scenario and considering both time slices, a strong decrease, in the species’ presence, is observed in southern Tagus river banks, which leads to conclude that a potential risk for the species’ natural habitat exists, mainly because it is known a putative refuge in the south (Serra de Monchique) [6]. The increase of suitable areas, on the northern bank of the Tagus river (central and north mountain areas and Lisbon region) will allow the species to spread towards the southwest-northeast of Portugal. However, this theoretical expectation requires the species to keep its migration pace during such a short period. In another study, the authors corroborate that even relatively moderate projections suggest that climate change has the potential to alter the different tree species distribution in the region [2], with a clear shift northwards.

Consequently, new species admixture and competition, including human interaction, are expected. Similarly to other studies worldwide (e.g. [39, 40]), we foresee both reductions and increases in range sizes, for the strawberry tree, depending on: 1. the region considered (north or south); 2. the intensity of the observed climate change, and: 3. the ability of the species to disperse. Central and northern mountainous regions are predictably suitable areas for *A*. *unedo*. The area’s suitability reduction in the south and the potential competition with agriculture and other occupational uses (e. g., others forest species, and pastures) in the north, are main concerns considering the future maintenance of the strawberry trees’ scrublands in these regions. Additionally, this species-area with increasing economic interest in Portugal may be severely reduced in the next decades, particularly in the south of the country.

The impact of climate change on biodiversity and alteration in species distribution might be drastic. The intensity of this impact will depend on the species’ ability to cope with landscape fragmentation, particularly in the Mediterranean region. The species’ dispersion is accelerated, even in fragmented areas, under long-distance events, but the effect on the landscape discontinuity increases when this phenomenon is less probable [41]. Mechanisms by which seeds may be transported to unusually large distances are diverse and include dispersal through birds activity, which is a common mechanism for *A*. *unedo* seed dispersal [11, 42]. The present day species’ distribution is likely to have been impacted by population fragmentation caused by past climatic oscillations [43], as well as more recent anthropogenic fragmentation and increasingly intense wildfires ([6] and references therein). In fragmented populations, particularly in marginal populations, the impact of climate change increases the risk of species’ genetic impoverishment, unless extensive gene flow exists among population [3]. The species’ survival depends on the speed of dispersal, especially in fragmented landscapes, to keep up with climate change [41]. Indeed, according to Santiso et al. [44], *A*. *unedo* reveals a considerable ability to disperse, migrating over thousands of kilometers, and this will be very helpful under future climate changes scenarios.

Additionally, populations respond to abrupt environmental change by evolving phenotypic plasticity and genetic evolution, but evolutionary adaptation may not be enough to assure the species’ survival under climate change [45]. The phenotypic plasticity means that a genotype shows different phenotypes under diverse environmental conditions, and a tree with higher phenotypic plasticity, such as *A*. *unedo*, may be submitted to less selection pressure, resulting in populations less prepared to face climate change [3]. Nevertheless, this decreased in selection might be compensated by the increased phenotypic match allowed by plasticity [46]. The strawberry tree has a high degree of phenotypic plasticity according to provenance trials results [47], e.g. in water availability [48], and plasticity might be one important factor for the species to endure the ongoing environmental changes. Nevertheless, under the pressure of changing environments, populations’ phenotypic plasticity responses may depend on the intensity and rapidness of the changes in environmental features and the amount of change to the phenotypic characteristics that individual populations might possess [3]. It is worthwhile noticing that, despite the dispersal mechanisms of this species, it will be a very short period to adapt and disperse, which may result in the implementation of additional conservation measures.

### Ecological niche suitability predictions for the Last Glacial Maximum and Mid-Holocene

For the Last Glacial Maximum the paleoclimatic model using Maxent and levels of genetic diversity from a previous study [6] showed a northern range and identifies few relatively small refugia followed by the expansion of the species later during the warm and humid period of the Holocene. A putative refugia in the northern part of the country was not supported by chloroplast DNA (cpDNA) data [6], since the sampled northern populations in that study had lower values of genetic diversity and number of haplotypes. Indeed, the results obtained in that study indicate refuges’ location in the southern part of the country and in the coastal center [6]. A cryptic refuge in the East-central mountain region is also referred in the literature [49]. The cpDNA is maternally inherited in *A*. *unedo* [6], which may result in less among population gene flow intermix of the initial genetic structure, established during survival in refuges and colonization. Together with low mutation rate, cpDNA is a very suitable source of markers to study the species in the late quaternary history, since the cpDNA is transmitted through seeds, which is likely to retain historical structures (past migration routes, colonization dynamics). Unlikely, biparental inherited nuclear markers, show a different behavior, where gene flow occurs both by seed and pollen transportation [50, 51]. Additionally, fossil charcoal records, in central Portugal (Estremadura region), support the presence of Mediterranean taxa, including *A*. *unedo*, during the Last Glacial Maximum (LGM) [16, 52]. Moreover, Santiso and colleagues [44] in a phylogeographic study of the species, refer that the strawberry tree survived the late quaternary, in the western Mediterranean, due to the existence of ancestral haplotypes, restricted to that region. Indeed, during the Pleistocene, the regions with lower latitudes in Europe were cooler, and the Iberian Peninsula coastal areas were less glaciated, compared to inland regions ([8] and references therein).

The species’ area shrank in the northern region probably due to change in climatic conditions and possible competition with other species, such as oaks, related with climatic conditions improvement in the Holocene. Temperate deciduous forests extended into regions that now have a Mediterranean-type climate, where the dominant vegetation today is either evergreen/warm mixed forest or xerophytic woods and scrub ([53] and references therein). Oak populations sizes grew rapidly as climate became warmer [54] and, as previously referred, owing to its poor canopy, *A*. *unedo* cannot compete successfully with *Quercus* and is eventually shaded out [11]. Indeed, in the Mid-Holocene the strawberry tree remained in sloppy areas in the northern region (Fig 2C and S2B Fig). In a palynological study made by López-Merino [55] in the NW Iberia, a clear increase of deciduous oaks is observed around the Mid-Holocene, and the increase of *Arbutus* population was only observed when the mature oak forest was substituted by an open oak forest with an increasing expansion of grass- and shrubland. In the putative refuge areas, southern part of the country and coastal center, the type of soil is generally basic, and oakwoods, such as *Quercus robur*, *Q*. *pyrenaica* and *Q*. *suber*, prefer acid soils [56], thus favoring the strawberry tree spread, due to its tolerance to soils with pH values from 4 to 7 [11].

The Mid-Holocene was a warm quaternary period, with rapid and drastic climatic changes that shaped forest evolution [8]. During the transition from the Late Pleistocene to the Holocene a significant increase of temperature was observed and higher values of rainfall were recorded in the region [16] and thermophilous taxa spread from cryptic refugia, such as *A*. *unedo*. Palaeobotanic evidence of scrublands, including *A*. *unedo* and other thermophiles shrubs, during the Mid-Holocene existed in the southern region, despite the fact that the species produces low amounts of pollen [57]. Additionally, fossil charcoal evidences, in central-western Portugal, indicated the presence of *A*. *unedo* in the MH [17].

The Mid-Holocene vegetation history reconstruction in east-central Portugal (Serra da Estrela), was done with a pollen diagram, using the sediments of a 1 400 m altitude lake. The authors stressed that the main factors for the changes in vegetation were due to climate change, in the initial phases and, gradually dominated by human activity, in the later phases [58]. The activities developed by humans have, since long, altered plant distribution, and forest clearing leads to communities’ of scrub-type, including Ericaceae [58, 59]. Given our model (Fig 1B), a probability, yet low, in for the presence of the species in east-central Portugal, exists, in the Mid-Holocene. Human activities might have triggered the species distribution in this region. According to Carrión et al. [59] and van der Knaap & van Leeuwen [58], the change in forest composition, in the Iberian Peninsula was due to climate change from xerothermic to mesothermic, as well as to growing anthropogenic influence, at least since the Mid-Holocene.

### Conclusions

The information retrieved in the current study provides information about the potential changes in the *A*. *unedo* distribution area, due to climate change, in the near future (2050 and 2070), considering two warming scenarios, and in the Last Glacial Maximum and Mid-Holocene. The distribution of the species in the LGM and MH agrees with previous genetic and fossils studies in the region. Two presumed refuges for the species were detected, possibly since the Last Glacial Maximum and predicted for the Mid-Holocene, together with a putative cryptic refuge in the east-central mountain region. The potential habitat envelope will decrease, for all the computed future scenarios, in the southern regions. The emergence of new suitable areas, in the central and northern mountains, will increase with climatic and topographic suitable spots, for the species. These changes, in the *A*. *unedo* potential habitat, induced by climate change, follow the expected pattern depicted by other authors for other species [2], in the region. Increase in altitude and latitude toward the North, and potential competition among species and farming is expected. Moreover, conservation strategies, integrating shifting climate space knowledge and additional studies in understanding the dispersal mechanisms, by which this species might achieve rapid large-scale migrations in the coming century, will be required. Additional concern about the species conservation, particularly in the southern putative refuge, must be stressed, and policy makers urged to assess this issue in face of the new global era.

## Supporting information

S1 Fig Representation of the seven bioclimatic variables used in the Maxent models for the *A*. *unedo* habitat suitability predictions in the different tested scenarios. (A) Present (current climate conditions). (B) Last Glacial Maximum, 22 000 BP. (C) Mid-Holocene, 6 000 BP. (D) Future 2050, RCP 4.5. (E) Future 2070, RCP 4.5. (F) Future 2050, RCP 8.5. (G) Future 2070, RCP 8.5.
(PDF)

S2 Fig Representation of the topographic variables used in the Maxent models for the *A*. *unedo* habitat suitability predictions. (A) Altitude. (B) Slope.
(PDF)

S3 Fig Response curves of the most influential environmental variables to MaxEnt models for strawberry tree in the present. (A) BIO15. (B) Slope. (C) Altitude (dem). (D) BIO2. (E) t_min_.
(PDF)

S1 Table Species’ presence geographical coordinates. The dataset with the 318 locations where the species was found present in the region of study, in a 1-Km^2^ grid level. Longitude (W) and latitude (N) in decimal degrees.
(XLSX)

S2 Table Variables extracted from WorldClim. The climatic variables code, name and dimensions.
(DOCX)

S3 Table Bayesian analysis results. Mutual information analysis from the Bayesian model built for the knowledge of the strawberry tree most influential bioclimatic variables (our unpublished data).
(DOCX)
