# Supplementary material for: Bioclimatic modeling in the Last Glacial Maximum, Mid-Holocene and facing future climatic changes in the strawberry tree (Arbutus unedo L.)
Source: PLoS One. 2019 Jan 9;14(1):e0210062. doi: 10.1371/journal.pone.0210062 (PMC6326469; doi:10.1371/journal.pone.0210062)

t_{max} - Monthly average maximum temperature

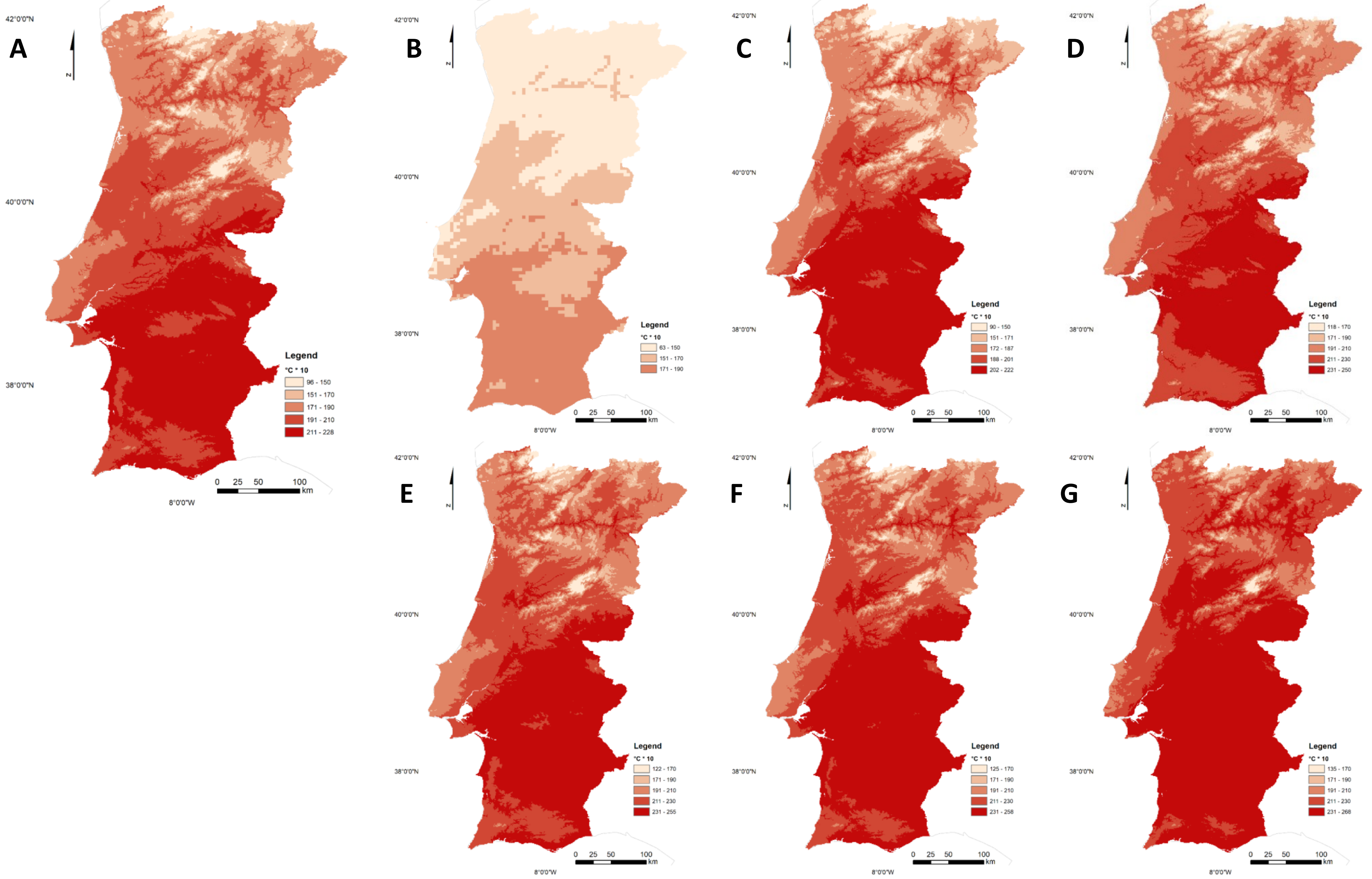

t_{min} - Monthly average minimum temperature

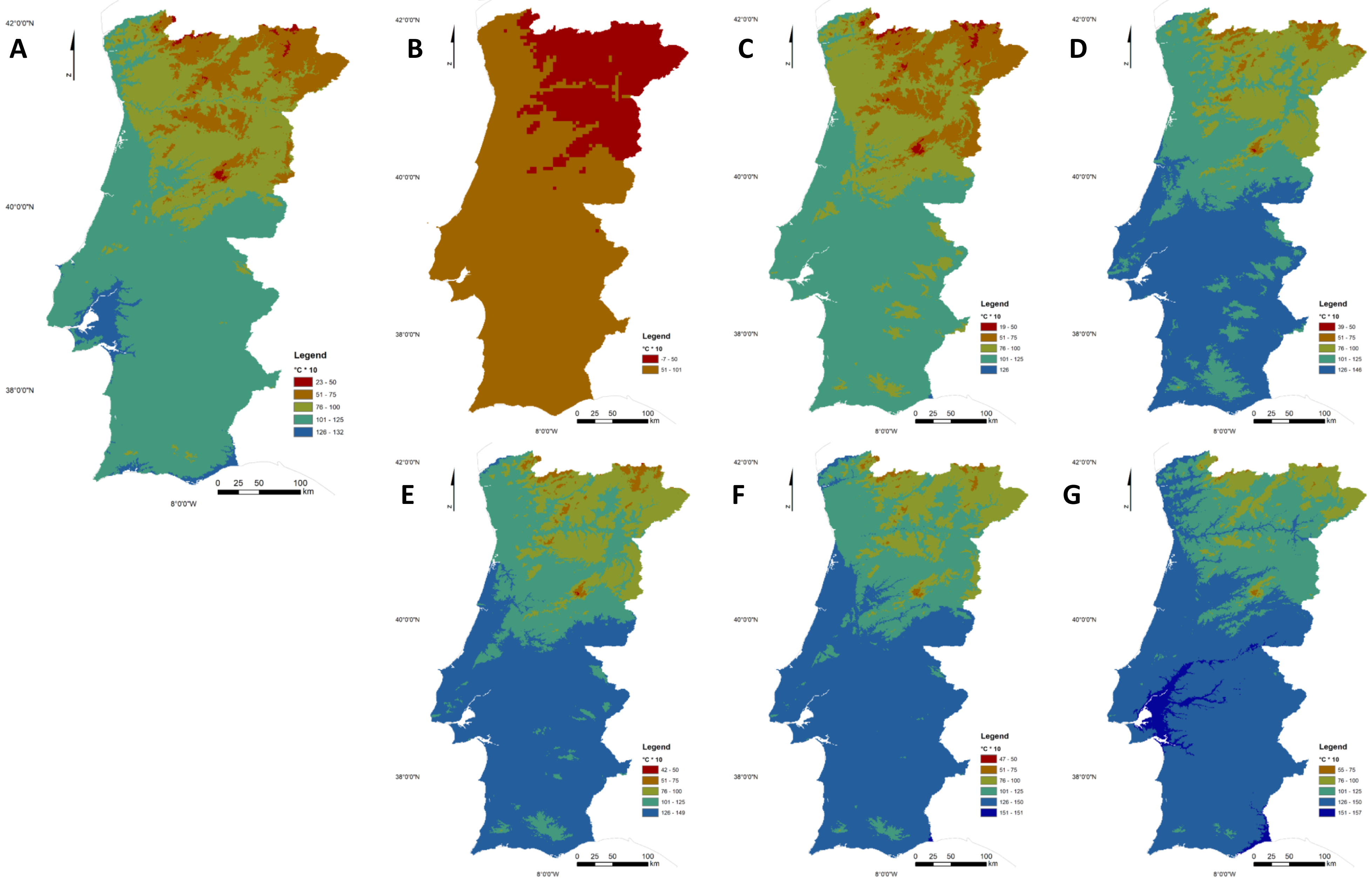

Bio1 - Annual mean temperature

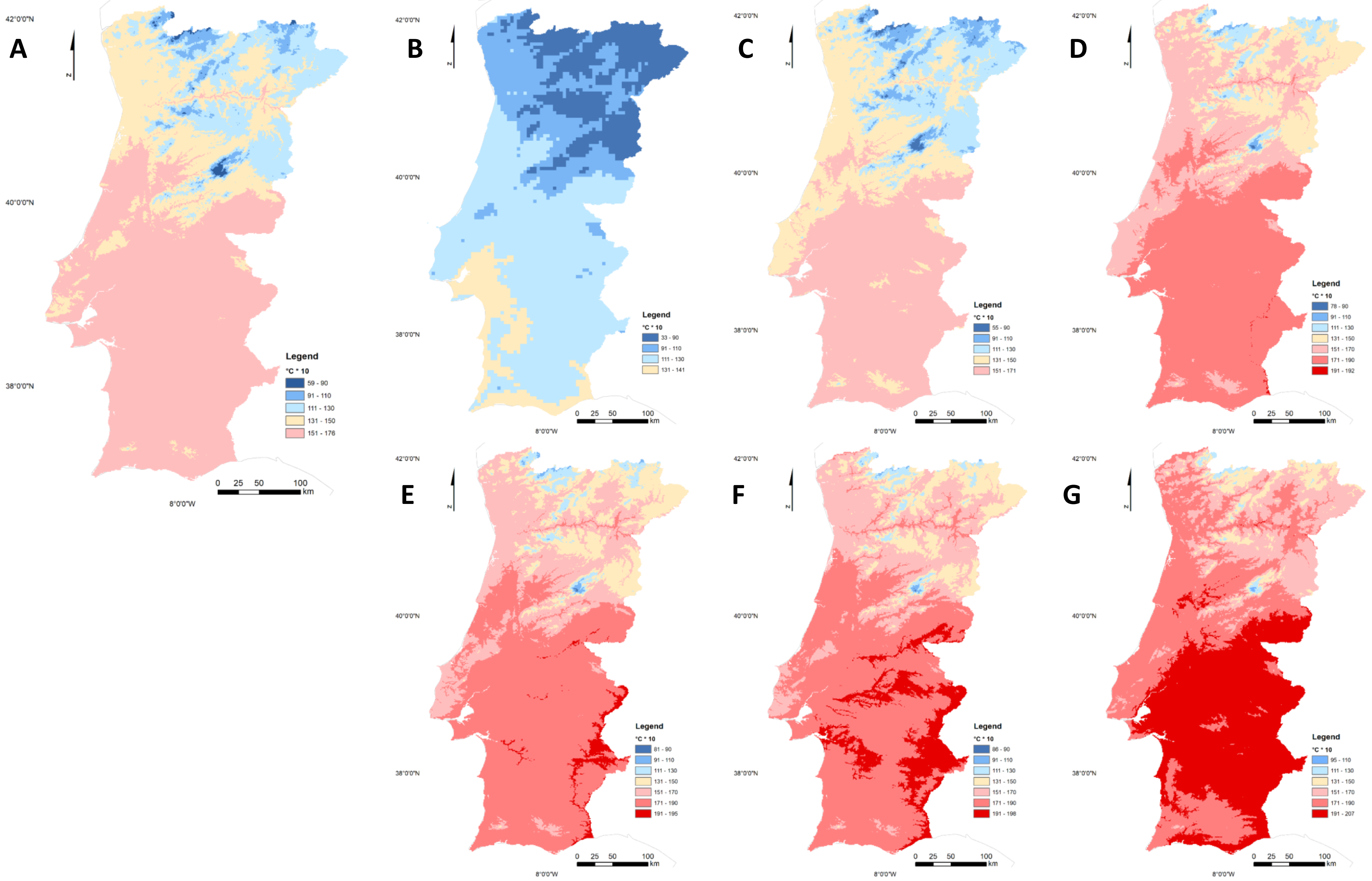

Bio2 - Mean diurnal range (mean of monthly (max temp - min temp))

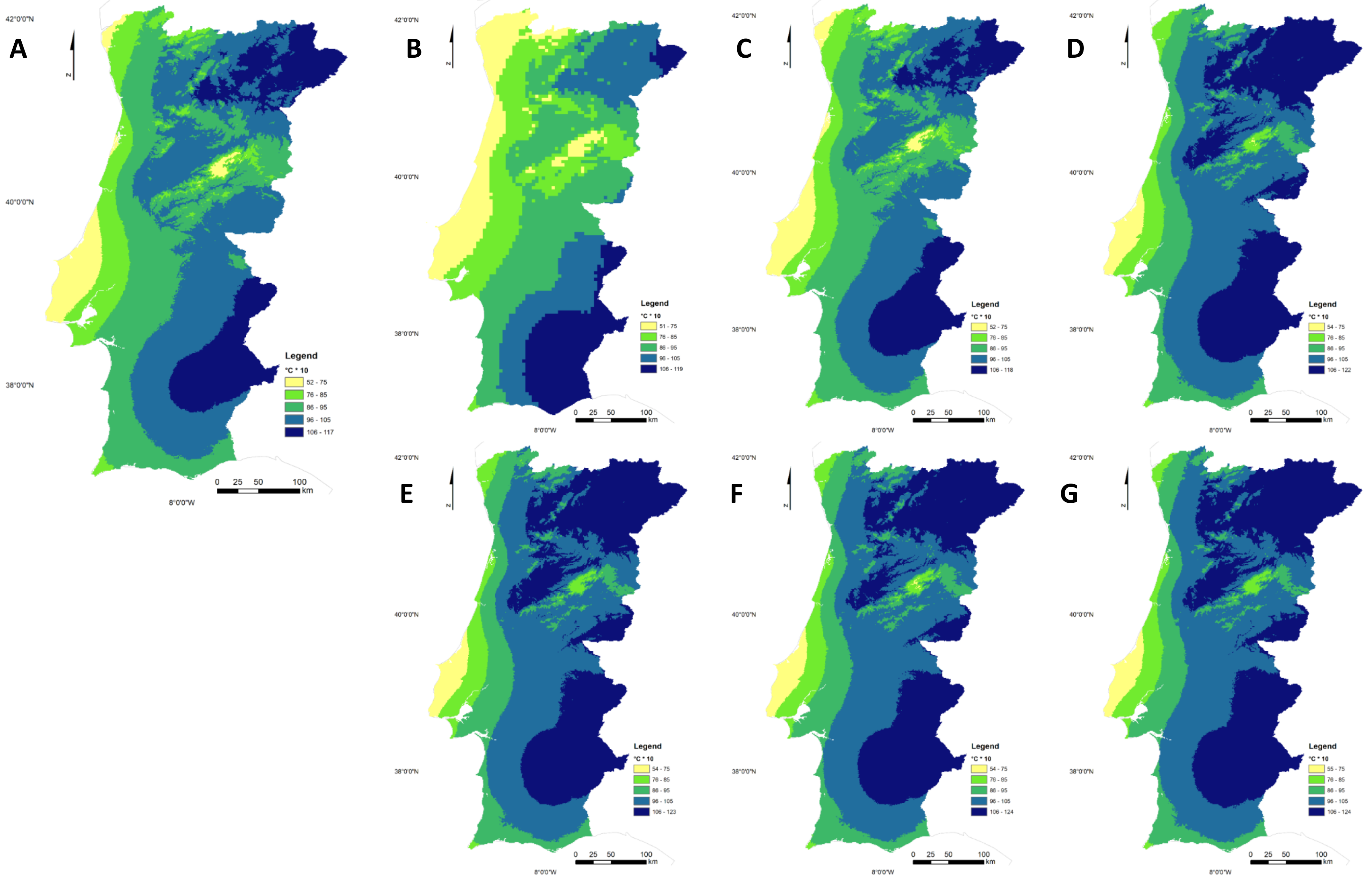

Bio5 - Max. temperature of warmest month

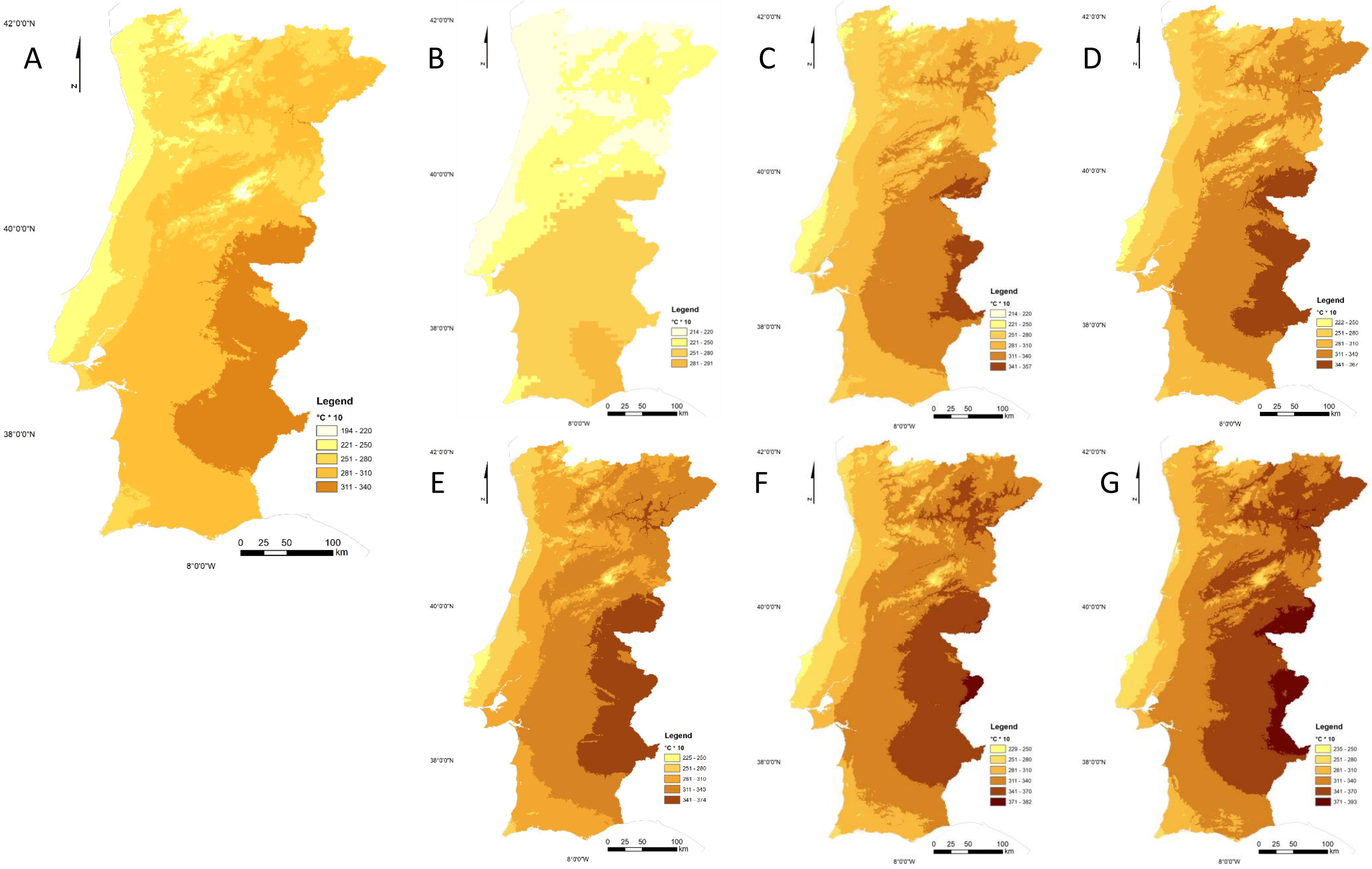

Bio9 - Mean temperature of driest quarter

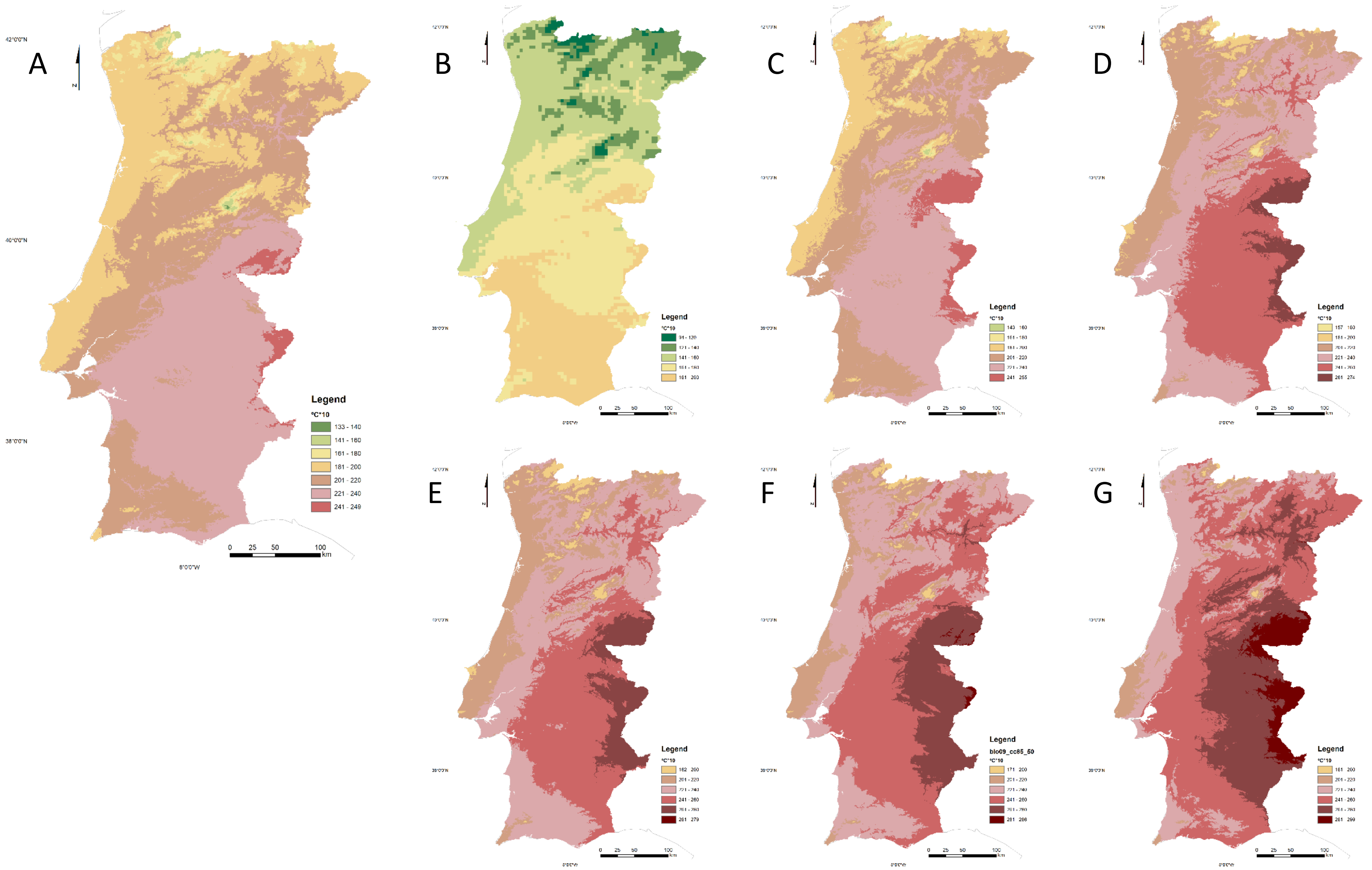

Bio15 - Precipitation seasonality (coefficient of variation)

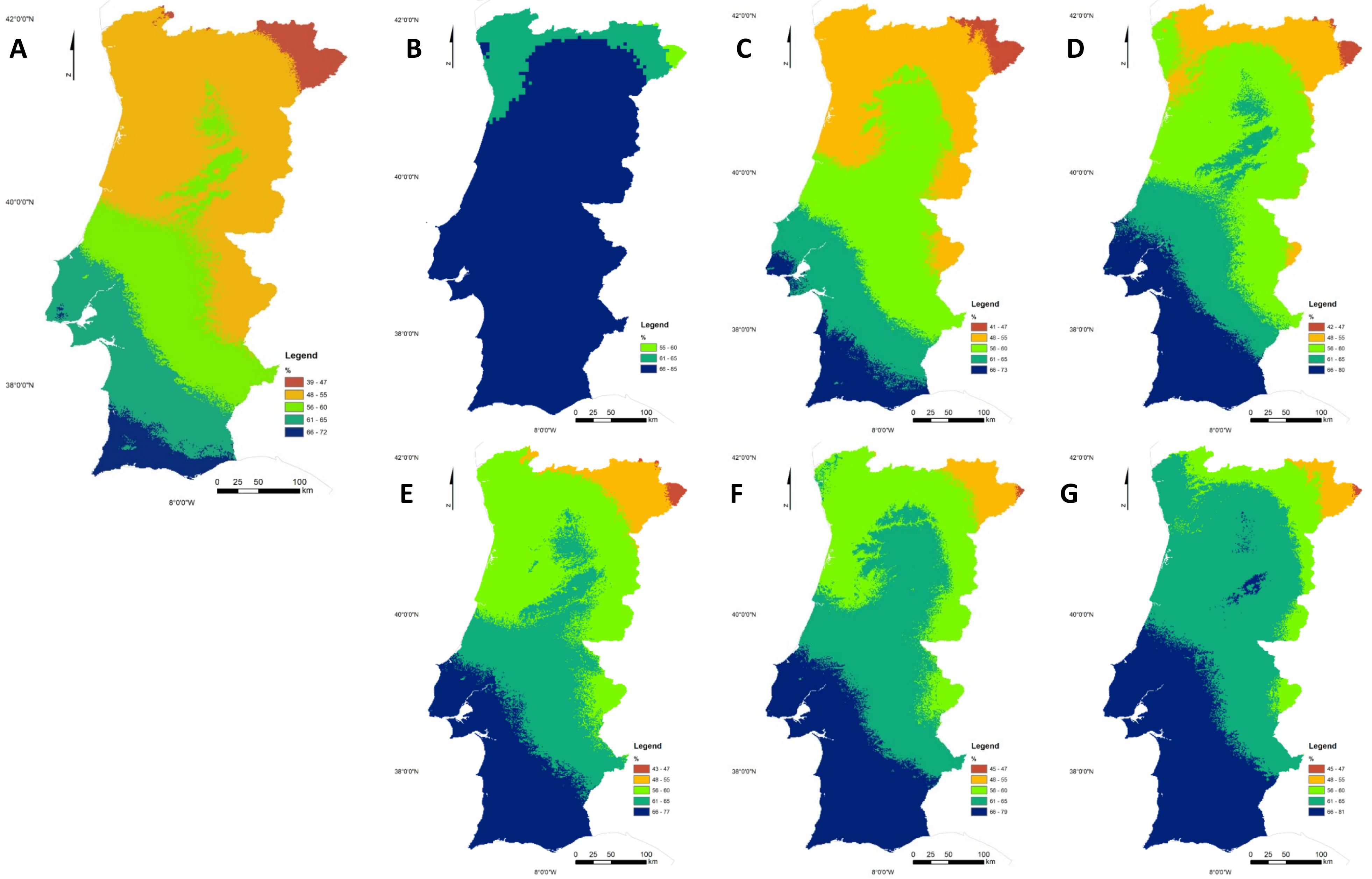

Supplement: S1 Fig — (A) Present (current climate conditions). (B) Last Glacial Maximum, 22 000 BP. (C) Mid-Holocene, 6 000 BP. (D) Future 2050, RCP 4.5. (E) Future 2070, RCP 4.5. (F) Future 2050, RCP 8.5. (G) Future 2070, RCP 8.5. (PDF) [file pone.0210062.s001.pdf]
