# Supplementary figures and images for: Bioclimatic modeling in the Last Glacial Maximum, Mid-Holocene and facing future climatic changes in the strawberry tree (Arbutus unedo L.)
Source: PLoS One. 2019 Jan 9;14(1):e0210062. doi: 10.1371/journal.pone.0210062 (PMC6326469; doi:10.1371/journal.pone.0210062)

**Altitude**

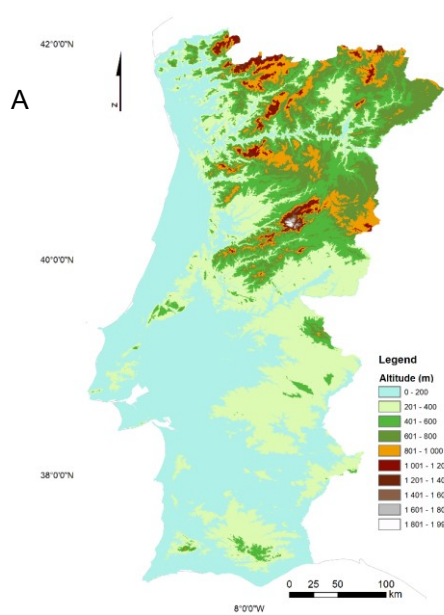

**Slope**

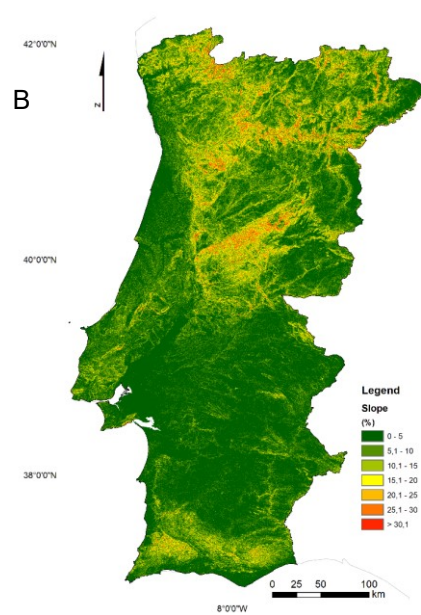

Supplement: S2 Fig — (A) Altitude. (B) Slope. (PDF) [file pone.0210062.s002.pdf]

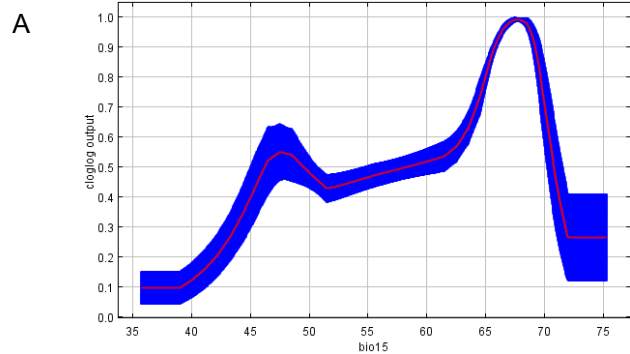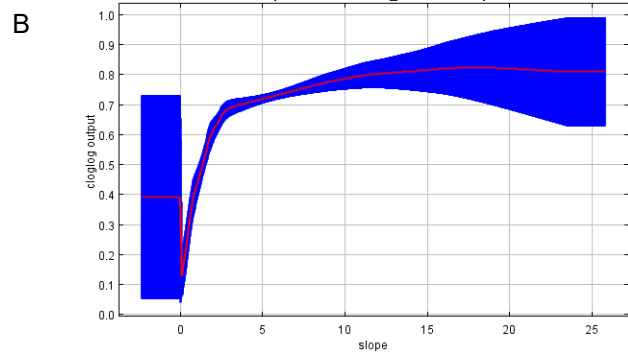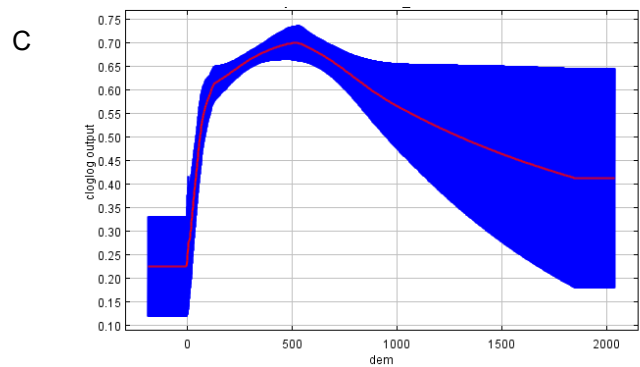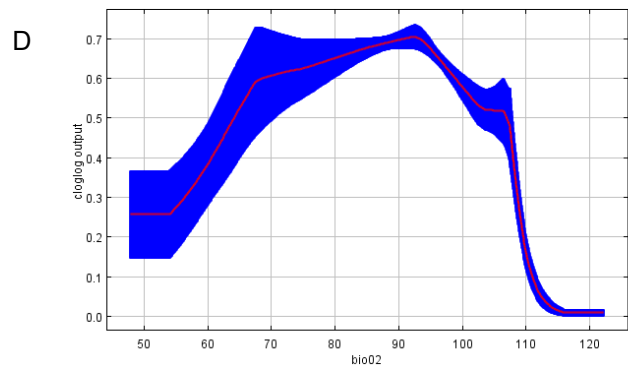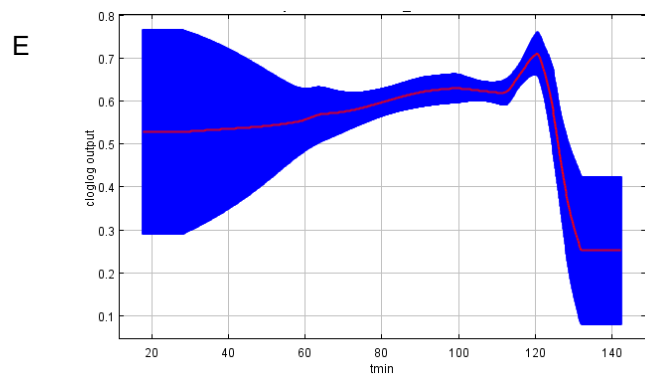

Supplement: S3 Fig — (A) BIO15. (B) Slope. (C) Altitude (dem). (D) BIO2. (E) tmin. (PDF) [file pone.0210062.s003.pdf]
