# Supplementary material for: Bioclimatic modeling in the Last Glacial Maximum, Mid-Holocene and facing future climatic changes in the strawberry tree (Arbutus unedo L.)
Source: PLoS One. 2019 Jan 9;14(1):e0210062. doi: 10.1371/journal.pone.0210062 (PMC6326469; doi:10.1371/journal.pone.0210062)
Supplement: S2 Table — The climatic variables code, name and dimensions. (DOCX) [file pone.0210062.s005.docx]

| **Code** | **Climate variable** | **Unities** |
| --- | --- | --- |
| t_max_ | Monthly average maximum temperature | °C * 10 |
| t_min_ | Monthly average minimum temperature | °C * 10 |
| BIO1 | Annual mean temperature | °C * 10 |
| BIO2 | Mean diurnal range (mean of monthly (max temp - min temp)) | °C * 10 |
| BIO3 | Isothermality (BIO2/BIO7) (* 100) | % |
| BIO4 | Temperature seasonality (standard deviation *100) | % |
| BIO5 | Max. temperature of warmest month | °C * 10 |
| BIO7 | Temperature annual range (BIO5-BIO6) | °C * 10 |
| BIO8 | Mean temperature of wettest quarter | °C * 10 |
| BIO9 | Mean temperature of driest quarter | °C * 10 |
| BIO10 | Mean temperature of warmest quarter | °C * 10 |
| BIO11 | Mean temperature of coldest quarter | °C * 10 |
| BIO12 | Annual precipitation | mm |
| BIO13 | Precipitation of wettest month | mm |
| BIO14 | Precipitation of driest month | mm |
| BIO15 | Precipitation seasonality (coefficient of variation) | % |
| BIO16 | Precipitation of wettest quarter | mm |
| BIO17 | Precipitation of driest quarter | mm |
| BIO18 | Precipitation of warmest quarter | mm |
| BIO19 | Precipitation of coldest quarter | mm |
