# Supplementary material for: Bioclimatic modeling in the Last Glacial Maximum, Mid-Holocene and facing future climatic changes in the strawberry tree (Arbutus unedo L.)
Source: PLoS One. 2019 Jan 9;14(1):e0210062. doi: 10.1371/journal.pone.0210062 (PMC6326469; doi:10.1371/journal.pone.0210062)
Supplement: S3 Table — Mutual information analysis from the Bayesian model built for the knowledge of the strawberry tree most influential bioclimatic variables (our unpublished data). (DOCX) [file pone.0210062.s006.docx]

|  | Mutual Information  (MI) | Relative Significance  (RS) | G-test | *p-value* |
| --- | --- | --- | --- | --- |
| ***BIO 15 Precipitation seasonality*** | ***0.000810*** | ***1.0000*** | ***71.1407*** | ***<0.01*** |
| ***BIO 5 Max. temperature of warmest month*** | ***0.000717*** | ***0.8852*** | ***62.9727*** | ***<0.01*** |
| ***BIO 9 Mean temperature of driest quarter*** | ***0.000643*** | ***0.7938*** | ***56.4734*** | ***<0.01*** |
| ***BIO 2 Mean diurnal range*** | ***0.000378*** | ***0.4667*** | ***33.1990*** | ***<0.01*** |
| ***Tmax Monthly average max. temperature*** | ***0.000247*** | ***0.3049*** | ***21.6935*** | ***<0.01*** |
| ***BIO 1 Annual mean temperature*** | ***0.000251*** | ***0.3099*** | ***22.0448*** | ***<0.01*** |
| ***Tmin Monthly average min. temperature*** | ***0.000189*** | ***0.2333*** | ***16.5995*** | ***<0.01*** |
| BIO 17 Precipitation of driest quarter | 0.000157 | 0.1938 | 13.7890 | 0.01 |
| BIO 18 Precipitation of warmest quarter | 0.000142 | 0.1753 | 12.4716 | 0.01 |
| BIO 4 Temperature seasonality | 0.000129 | 0.1593 | 11.3298 | 0.01 |
| BIO 7 Temperature annual range | 0.000111 | 0.1370 | 9.7489 | 0.01 |
| BIO 11 Mean temperature of coldest quarter | 0.000103 | 0.1272 | 9.0463 | 0.02 |
| BIO 16 Precipitation of wettest quarter | 0.000087 | 0.1074 | 7.6410 | 0.03 |
| BIO 12 Annual precipitation | 0.000084 | 0.1037 | 7.3776 | 0.03 |
| BIO 14 Precipitation of driest month | 0.000083 | 0.1025 | 7.2897 | 0.03 |
| BIO 13 Precipitation of wettest month | 0.000082 | 0.1012 | 7.2019 | 0.05 |
| BIO 10 Mean temperature of warmest quarter | 0.000074 | 0.0914 | 6.4993 | 0.06 |
| BIO 19 Precipitation of coldest quarter | 0.000068 | 0.0840 | 5.9723 | 0.07 |
| Bio 8 Mean temperature of wettest quarter | 0.000061 | 0.0753 | 5.3575 | 0.07 |
| Bio 3 Isothermality | 0.000049 | 0.0605 | 4.3036 | 0.10 |
| Bio 6 Min temperature of coldest month | 0.000037 | 0.0457 | 3.2496 | 0.33 |
